# Phytogenic Selenium Nanoparticles Elicited the Physiological, Biochemical, and Antioxidant Defense System Amelioration of Huanglongbing-Infected ‘Kinnow’ Mandarin Plants

**DOI:** 10.3390/nano12030356

**Published:** 2022-01-22

**Authors:** Muhammad Ikram, Naveed Iqbal Raja, Zia-Ur-Rehman Mashwani, Ahmad Alsayed Omar, Azza H. Mohamed, Seema Hassan Satti, Efat Zohra

**Affiliations:** 1 Department of Botany, PMAS Arid Agriculture University, Rawalpindi 46300, Pakistan; mashwani@uaar.edu.pk (Z.-U.-R.M.); Seemasatti@gmail.com (S.H.S.); iffatzohra003@gmail.com (E.Z.); 2 Biochemistry Department, Faculty of Agriculture, Zagazig University, Zagazig 44519, Egypt; 3 Citrus Research and Education Center, University of Florida, IFAS, Lake Alfred, FL 33850, USA; 4 Agricultural Chemistry Department, Faculty of Agriculture, Mansoura University, Mansoura 33516, Egypt

**Keywords:** citrus, selenium nanoparticles, huanglongbing, phyto-nanobiotechnology, antioxidant system, *Candidatus* Liberibacter asiaticus

## Abstract

Citrus greening or huanglongbing (HLB) is commonly known as yellow dragon disease and affects citrus production worldwide. Therefore, it has a significant impact on and deleterious effects in the agro-industrial sector. Significant efforts have been made to combat this disease and mitigate its destructive impact on citrus production, but still, there is no effective biocompatible treatment available to control HLB disorder. This study is considered the first biocompatible approach to evaluate the potential of phytogenic selenium nanoparticles (SeNPs) to improve the health of HLB-infected ‘Kinnow’ mandarin plants. Polymerase chain reactions (PCRs) with specific primers were used to detect HLB disease in ‘Kinnow’ mandarin plants, and PCR products were sequenced to identify *Candidatus* Liberibacter asiaticus (CLas), and accession numbers for CLas1 and CLas2, MZ851933 and MZ851934, respectively, were obtained. SeNPs were synthesized by using *Allium sativum* L. clove extract as a reducing, capping, and stabilizing agent and various techniques such as UV-visible spectrophotometry, energy dispersive X-rays, scanning electron microscopy (SEM), Fourier transformed infrared spectroscopy (FTIR), and X-ray diffraction analysis (XRD) were used to confirm the biogenesis of SeNPs. Different concentrations of SeNPs (25, 50, 75, and 100 mg L^−1^) were exogenously applied to HLB-infected ‘Kinnow’ mandarin plants and obtained spectacular results. The obtained results from the current study proved that 75 mg L^−1^ of SeNPs was most effective to improve the chlorophyll, carotenoids, relative water content (RWC), membrane stability index (MSI), total soluble sugar (TSS), superoxide dismutase (SOD), peroxidase (POD), catalase (CAT), total flavonoid content (TFC), and total phenolic content (TPC) and significant decrease was observed in hydrogen peroxide (H_2_O_2_), malondialdehyde (MDA), and proline (PRO) contents of HLB-infected ‘Kinnow’ mandarin plants as compared to untreated diseased citrus plants. In conclusion, these results allow us to synthesize the SeNPs formulation as a promising management strategy to treat the HLB disease in citrus plants.

## 1. Introduction

Citrus belongs to the family Rutaceae. It has a prominent position among various fruiting crops worldwide. Citrus fruits are cultivated in subtropical and tropical environments between latitude 35° N and 35° S [1]. Citrus fruits are consumed in large quantities worldwide because of their notable nutritional status and bioactives that have health-promoting effects. Citrus fruits contain bioactives such as carotenoids, flavonoids, essential oils, limonoids, and synephrine, which protect from various disorders, including cancer, cardiovascular, digestive, and inflammatory diseases [2,3,4]. In addition, citrus fruits possess an important position worldwide because of their ascorbic acid content, dietary fibers, and carbohydrates such as fructose, sucrose, and glucose contents which decrease cholesterol levels and consequently mitigate devastating problems of the digestive system. Citrus fruits play an essential role in human health and protect from various chronic maladies [5]. Citrus is predominantly cultivated in the province of Punjab and is one of the chiefly exported fruits of Pakistan [6]. In recent decades, citrus has been vulnerable to various diseases caused by viruses, bacteria, fungi, and nematodes [7]. Huanglongbing (HLB) or yellow dragon disease is currently the most destructive citrus disease, devastating the citrus industry worldwide [8]. HLB is a bacterial disease that is drastically decreasing citrus production worldwide. HLB is caused by the un-culturable phloem limited fastidious α-proteobacterium *Candidatus* Liberibacter spp.—having three casual organisms, though the *Candidatus* Liberibacter asiaticus is the most common in America and Asia—which limits the uptake and movements of essential nutrients in the phloem, ultimately affecting the citrus tree health and fruit quality [9,10]. The bacterium first moves from infected sites toward the roots and multiplies, ultimately damaging the roots first and then the leaves. In addition, after multiplying, the bacterium moves upward and damages the phloem cells, resulting in blockages due to the deposition of proteins and callose, ultimately affecting the tree’s health [11]. Typical early diagnostic symptoms of HLB-infected citrus plants are flower drop, leaf loss, and the yellowing of isolated shoots. HLB-infected citrus plants produce misshapen, underdeveloped, and green unmarketable citrus fruits with aborted seeds [9,12]. In Pakistan, HLB disease has been proven to be the major cause of citrus decline, and it results in massive losses to the citrus industry, particularly in the Sargodha, Multan, and Malakand districts of Punjab and Khyber Pakhtunkhwa, respectively [13]. In Pakistan, HLB disease incidence ranges between 4.6 and 24% among the ‘Mosambi’ and ‘Kinnow’ mandarin plants. In the recent past, it has also been reported that HLB is a major threat to citriculture among distinguished tehsils of the district of Sargodha, i.e., Bhera, Bhalwal, Shahpur, Kotmomin, Sillanwali, and Sahiwal [14]. There is currently no proper cure, and unique managing strategies have not been established to cure HLB disease.

It is well known that HLB disease spreads by Asian citrus psyllids (*Diaphorina citri* Kuwayama), so various insecticides are commonly being used to reduce disease severity and disease incidence in commercial citrus. However, the use of insecticides as a major controlling strategy is not an eco-friendly and sustainable approach because of its serious side effects, including environmental pollution, reduction in natural enemies, and the eruption of secondary pest outbreaks [15]. Although, the use of insecticides is considered a notable and practical approach to reduce citrus psyllid populations. Alternative approaches emphasize the identification and fabrication of novel, potentially effective, and biocompatible antimicrobial agents against the bacteria. During the past decades, antimicrobial agents were over-exploited, and various antibiotics have lost their effectiveness [16]. The extensive use and misuse of antibiotics against bacteria exacerbate high pressure on the pathogen that develops serious antibiotic resistance. The best results were acquired with β-lactams and tetracyclines; however, the emergence of microbial resistance and several indirect side effects on human health is a growing and eminent concern that limits the use of antibiotics at a field scale [17,18].

Consequently, there is an urgent need to find a sustainable solution to treat HLB disease with minimal side effects. In this scenario, green nanotechnology has provided the most effective and safe strategy to minimize the detrimental impact of pathogens on various crops and fruits. Nanobiotechnology is an emerging biofertilizer factory used in the agriculture sector, mitigating agrochemical usage, increasing microbial resistance in plants, and enhancing nutrient uptake, utilization, and plant growth. Recently, various nanoparticles were explored to improve the quality and quantity of crops such as cucumber, wheat, tomato, corn, and citrus under environmental stresses [19,20]. Selenium is an essential element required for plants and animals’ normal physiological and biochemical functioning at low concentrations, but it becomes harmful and toxic at higher doses. Among various nanomaterials, plant-based selenium nanoparticles (SeNPs) have appeared as excellent antimicrobial and antioxidant agents to mitigate plant pathogens’ deleterious effects in various crops [21]. SeNPs synthesized from plant extract have been shown to have outstanding biocompatibility, bioavailability, less toxicity, biodegradability, and an environmentally friendly nature [22]. This is because the plant extract contains phenolic acid, cinnamic acid, tannin sesquiterpenes, and monoterpenes secondary metabolites, which act as both reducing agents and stabilize capping in the fabrication of SeNPs, which makes them biocompatible in nature [22]. Some studies also demonstrated that phytogenic SeNPs are 20-fold faster than bulk selenium [23]. It has been reported that SeNPs are effective against both Gram-negative and Gram-positive bacteria, but SeNPs are more effective against Gram-negative bacteria owing to their thin peptidoglycan layer [24]. The exact mechanism of plant-mediated SeNPs is still unknown, but some studies have reported that SeNPs with spherical shape and smaller size had easy access into the microorganism’s cells. It has been demonstrated that the antibacterial potential of SeNPs may be due to many reasons, such as the generation of reactive oxygen species (ROS) that are involved in the disruption of cell membrane integrity [25,26]. Some other studies have also reported that SeNPs inhibit the growth of bacteria by breaking the cell wall and then binding with the plasma membrane and altering the food metabolism and protein synthesis cycle, ultimately binding with the thiol groups present in the membrane proteins and causing the denaturation of bacterial cells, eventually leading to cell death [27]. Plant-mediated SeNPs are also very effective against human and plant pathogens.

In recent research studies, it is also reported that SeNPs are very useful against several diseases such as tomato leaf blight caused by *Alternaria alternata*, late blight of tomato caused by *Phytopthora infestan*, downy mildew in pearl millet caused by *Sclerospora graminicola* [28,29]. El-Saadony et al. [30] revealed that biological SeNPs are very effective against root rot and crown diseases caused by Fusarium species in wheat crops. Nanoparticles electrostatically interact with the cell wall and plasma membrane, which causes DNA destruction, ultimately leading to cell death [31]. In the present study, SeNPs were phyto-fabricated by garlic clove extract (*Allium sativum* L.). Garlic has outstanding therapeutic applications, including anti-cancer, hypoglycemic, antibiotic, and anti-inflammatory effects. Furthermore, some of the literature has suggested that garlic cloves have an array of therapeutic benefits, with antifungal, antibacterial, and antiviral properties [32]. Hence, it is reasonable to infer that the selenium nanoparticles capped and stabilized with essential metabolites present in garlic cloves extract may represent a nanoparticle with remarkable antimicrobial significance.

Currently, there is no scientific study reporting the application of plant-mediated SeNPs for combating HLB disease in citrus plants. The current work is the first study to show that SeNPs have a differential impact on the physiological, biochemical, and antioxidant defense system of HLB-infected ‘Kinnow’ mandarin plants. Various concentrations of SeNPs were exogenously applied to HLB-infected ‘Kinnow’ mandarin plants to identify the more effective concentration for treating HLB disease in citrus plants.

## 2. Materials and Methods

### 2.1. Research Area, Conditions and Selection of Specimens

For this study, 7-year-old ‘Kinnow’ mandarin trees, a hybrid of ‘King’ tangor (*Citrus nobilis*) × ‘Willowleaf’ mandarin (*Citrus deliciosa*) trees with severe HLB symptoms were selected from the village of Melowal, geographically located in the district of Sargodha, the tehsil of Bhera, the province of Punjab, Pakistan, situated at latitude 32.447259° N and longitude 72.031278° E. The trees were selected during the winter season in January 2020. The ‘Kinnow’ mandarin trees selected for the study were separated from the rest of the citrus trees by using a red ribbon along with a code number. Moreover, three branches from each of the trees under study were identified by using yellow ribbons to assess the changes induced by the SeNPs application.

### 2.2. Sample Collection, DNA Extraction, and Polymerase Chain Reaction (PCR) Protocol

HLB-affected leaves from 20 infected trees were collected to confirm the causative agent of HLB through PCR analysis. The HLB-affected leaves were picked from labeled tree branches with 7 to 11 leaves. The Cetyl Trimethylammonium Bromide (CTAB) protocol was used to extract the genomic DNA from the midrib of the infected leaves [33]. A fine powder of leaf midribs (250 mg) was obtained by grinding in liquid nitrogen, and the powder was mixed with 2% CTAB buffer (2.5 mL). Furthermore, the solution was centrifuged at 12,000 rpm for 10 min, the supernatant was collected, and then 0.5 mL of chloroform: isoamyl alcohol mixture (24:1) was added and centrifuged again at 12,000 rpm for 10 min. The upper layer was transferred to centrifuge tubes, and DNA was precipitated by adding an equal volume of isopropanol and then centrifuged at 12,000 rpm for 15 min. Moreover, 70% ethanol was used to wash the pellets twice, followed by drying and re-suspension in 100 µL of TE Buffer [34,35].

For the PCR protocol, forward primer A2 (5′-TATAAAGGTTGACCTTTCGAGTTT-3′) and reverse primer J5 (5′-ACAAAAGCAGAAATAGCA CGAACAA-3′) from outer membrane protein region rplKAJL-rpoBC operon (β-operon) were used. The reaction mixture for the PCR was carried out in a Thermal Cycler (T100^TM^ Thermal Cycler Singapore) by using 25 µL of 1 µM of each primer, 0.2 mM of each of the four dNTPs, 1X PCR buffer, 2.5 mM MgCl_2_, 0.5 units *Taq* DNA polymerase (Thermo Fisher Scientific, Waltham, MA, USA) and 1 µL DNA template. Furthermore, the amplification program was as follows: one cycle at 94 °C for 3 min; 35 cycles each at 92 °C for 45 s, 62 °C for 45 s and 72 °C for 90 s, followed by a 72 °C extension for 5 min [36]. The polymerase chain reaction product was visualized on 1.5% agarose gel. Furthermore, the HLB-infected ‘Kinnow’ mandarin plants were selected for further study.

### 2.3. Sequencing of PCR Products for Confirmation of Candidatus Liberibacter Asiaticus

The purification of PCR products from the primers A2/J5 was performed using JET Quick, Gel Extraction Spin Kit (GenoMed, Leesburg, FL, USA) sequenced on an automated sequencer. Moreover, DNA sequencing was compared to the NCBI (National Center for Biotechnology Information) database using BLAST. Furthermore, the obtained sequence was compared with the existing NCBI gene bank database and deposited under the accession numbers MZ851933 and MZ851934 for Clas1 and Clas2, respectively.

### 2.4. SeNPs Preparation and Characterization

#### 2.4.1. Preparation of Garlic Cloves Extract

Fresh garlic cloves (10 g) were collected from the local market of Rawalpindi, Punjab, Pakistan. The outer layer of the cloves was peeled out, dipped in freshwater, washed with distilled water to remove all the dust particles, and finely crushed using a pestle and mortar. The crushed cloves were macerated into 30 mL of Tris-HCl to maintain the mixture at pH 7.5. After that, the crushed cloves were centrifuged at 15,000 rpm for 15 min at room temperature. The supernatant was transferred to clean sterile centrifuge tubes and refrigerated to synthesize SeNPs [37,38].

#### 2.4.2. Bio-Fabrication of SeNPs

About 10 mL of the homogenized extract of garlic cloves was added dropwise to 20 mL of 10 mM sodium selenite solution followed by constant stirring for seven hours at room temperature for the biogenesis of SeNPs. The color of the solution changed to brick red, which indicated the synthesis of nanoparticles. The solution was subjected to centrifugation at 15,000 rpm for 10 min to obtain purified SeNPs. The obtained pellet was dried through a SpeedVac concentrator (SPD 120, Thermo Fisher Scientific) and kept at 4 °C for further use [39].

#### 2.4.3. Spectrophotometric Analysis of SeNPs

The bio-fabrication of SeNPs was confirmed using a UV-vis spectrophotometer (Labomed UVD 3500, Los Angeles, CA, USA). The analysis was performed by recording the UV-vis spectrum in the range of 200 to 800 nm [37].

#### 2.4.4. Scanning Electron Microscopic (SEM) Analysis of SeNPs

The morphological analysis of biofabricated SeNPs was performed through the scanning electron microscopic (SEM) technique. The SEM SIGMA (TESCAN MIRA3 FEG-SEM, NanoImages, LLC, CA, USA) model was used at 5 kV, and magnification was up to ×10 k. Furthermore, the sample was prepared by the drop-coating method on a carbon-coated copper grid. The resulting sample was dried by putting it under a mercury lamp for seven minutes, and blotting paper was used to remove the extra solution. The surface image of the bio-fabricated SeNPs was examined at different magnifications [40]. The Scherrer formula D = Kλ/(β cos θ) was used to predict the size of the SeNPs.

#### 2.4.5. X-ray Diffraction Analysis (XRD)

The XRD (X-ray diffraction analysis) of the SeNPs was examined by using a Siefert X-ray diffractometer operated at 40 kV and 30 mA within a 2θ area between 20 and 80° with intensity Cu-Kα radiation (λ = 0.15406 nm).

#### 2.4.6. Energy-Dispersive X-ray Spectroscopic Analysis (EDX) of SeNPs

The EDX instrument determined the elemental analysis of the biofabricated SeNPs. The samples’ preparation and analysis were carried out as described previously [41].

#### 2.4.7. Fourier Transform Infrared Spectroscopy (FTIR)

FTIR is an important analysis to study the various functional groups that are involved in the green synthesis of SeNPs by garlic clove extract. Dried SeNPs powder was pelleted with the potassium bromide and the FTIR spectrum (Perkin-Elmer FTIR-Spectrum, Akron, OH, USA) was obtained as a wavenumber in the range of 500–4000 cm^−1^.

### 2.5. Selection and Application of Biogenic SeNPs and Experimental Plan

To assess the effect of various concentrations of biogenic SeNPs, 18 HLB-infected ‘Kinnow’ mandarin trees were selected for the SeNPs exposure. A randomized complete block design (RCBD) was used with six different treatments along with three replicates. Different concentrations of biofabricated SeNPs were chosen for foliar applications on HLB-infected ‘Kinnow’ mandarin trees. Foliar applications were carried out with a sprayer machine (Hand Sprayer AP-20P made in China). The solution was sprayed at 4:00 a.m. and 9 a.m. to make sure the stomata were opening. All the treatments were applied exogenously two times with 15 days intervals before the February (2020) flowering stage. A detailed treatment plan is given below in Table 1.

### 2.6. Measurement of Physiological Parameters

#### 2.6.1. Chlorophyll a, Chlorophyll b, Total Chlorophyll, and Carotenoids Content

Chlorophyll a, b, and total chlorophyll contents were measured by taking into consideration the protocol used previously [42,43]. Fresh leaves (1 g) from each treatment were cut into small pieces and ground thoroughly with a pestle and mortar. Furthermore, 20 mL of 80% acetone and 0.5 g of MgCO_3_ were added and further ground following the method of Kamble et al. [42]. After that, the resulting mixture was incubated at 4 °C for 3 h, then the mixture was centrifuged at 2600 rpm for five minutes. The supernatant was transferred to a 100 mL volumetric flask, and then the volume was made up to 100 mL with the addition of 80% acetone. Further, the resulting solution was used for chlorophyll determination. The absorbance of each treatment sample was recorded at 480, 645 nm, and 663 nm using a spectrophotometer (Labomed UVD 3500, Los Angeles, CA, USA), using 80% acetone solution in a cuvette as a blank. The chlorophyll a, b, total chlorophyll (a + b), and carotenoid content were calculated by applying the following equations [44,45]:

Chlorophyll a (mg g^−1^) = 12.7 (A_663_) − 2.69 (A_645_) × V/1000 × W (1)

Chlorophyll b (mg g^−1^) = 22.9 (A_645_) − 4.68 (A_663_) × V/1000 × W (2)

Total chlorophyll (mg g^−1^) = 20.2 (A_645_) + 8.02 (A_663_) × V/1000 × W (3)

Carotenoid (mg g^−1^) = [A_480_ + (0.114 (A_663_) − (0.638 − A_645_)] × V/1000 × W (4)

where

A = Absorbance obtained at a specific wavelength;
V = Final volume of Chl extract in 80% acetone;
W = Weight of fresh tissue extracted.

#### 2.6.2. Estimation of the Relative Water Content (RWC)

The fresh leaves of ‘Kinnow’ mandarin plants were collected, and the fresh weights of all leaves were recorded individually. After that, the leaves were dipped in distilled water for 24 h and then the turgid weights of each sample were recorded individually. After this, the leaves were subjected to 65 °C for 72 h in the oven for drying, and ultimately the dry weights of the leaves were measured by using the following formula [46]:

RWC = (Fresh weight − Dry weight)/(Saturated weight − Dry weight) × 100(5)

#### 2.6.3. Estimation of Membrane Stability Index (MSI)

The MSI of treated and un-treated ‘Kinnow’ mandarin leaves was estimated by following the protocol used by Sairam [47]: 100 mg of fresh leaves were added to a small disc, washed, and then heated at 40 °C for 30 min in a water bath with 8 mL of distilled water. Furthermore, the electrical conductivity (C1) was also measured using an EC meter (HI98129 by Woonsocket, RI, USA, made in Romania). Thereafter, the discs along with samples were again subjected to boiling in water for 10 min, and the EC was recorded again (C2). The MSI was estimated using the given equation:

MSI = [1 − (C1/C2)] × 100(6)

#### 2.6.4. Estimation of Peroxidase (POD), Superoxide Dismutase (SOD), and Catalase (CAT)

The POD activity was assessed in the treated and un-treated citrus leaf samples by using a spectrophotometer at 470 nm using Guaiacol as a substrate according to the methodology of Denaxa et al. [48]. SOD activity was examined by considering its potential to avoid the photochemical reduction in nitro-blue-tetrazolium (NBT) by using the method described by Fimognari et al. [49]. The SOD activity was assayed by using a spectrophotometer at 560 nm. One unit of SOD activity was determined by the enzyme sum, resulting in a 50% inhibition of NBT photoreduction. The CAT activity of treated and un-treated ‘Kinnow’ mandarin plants was assayed by using the methodology described by Aebi [50].

#### 2.6.5. Extraction of Flavonoid and Phenolic Contents from ‘Kinnow’ Mandarin Plants Tissues

Citrus leaves were collected from labeled branches in each treatment to extract flavonoid and phenolic contents. The leaves were soaked, and a mortar and pestle were used for grinding in liquid nitrogen. One hundred mg of the homogenized sample was then added to a centrifuge tube. After that, methanol (1 mL) was added to the sample and vortexed for 15 min at 20 °C at 750 rpm. Then, the sample was sonicated for 15 min. After this, the samples were centrifuged at 12,000 rpm for 15 min. Finally, the resultant supernatant was collected and stored at −20 °C for further analysis [51].

##### Total Phenolic Content (TPC)

The TPC was measured by using the Folin–Ciocalteau reagent [52]. Furthermore, the reaction mixture was prepared by adding plant extract or Gallic acid standard (20 µL) and 0.9 mL of Folin–Ciocalteau reagent (10%), followed by the addition of 0.6 mL of sodium carbonate (7.5%, *w*/*v*) solution. The solution was allowed to stand for 60 min at room temperature, and then the absorbance was recorded at 760 nm. Then, the Gallic acid aqueous solution of a known concentration was used for calibration in the range of 100–600 ppm. The results were expressed as mg Gallic acid equivalents (GAE) g^−1^ FW.

##### Total Flavonoids Content (TFC)

The estimation of the TFC was quantified by following the colorimetric assay of Aktumsek et al. [53] using aluminum chloride. For this purpose, in a test tube, 50 µL of leaf extract was mixed with distilled water (200 µL). Then, 15 µL of 5% sodium nitrite solution and 15 µL of 10% aluminum chloride were added to the sample. Thereafter, all the test tubes were kept at room temperature for almost 5 min and 100 µL of 1 M sodium hydroxide and 1.2 mL of distilled water were added. The mixture was vortexed, and the absorbance of the resulting pink color was assessed using a spectrophotometer at 510 nm. The catechin aqueous solutions of known concentrations were used for calibration in a range of 10–200 ppm and all the results were expressed as mg catechin equivalents CEQ g^−1^ FW sample.

#### 2.6.6. Estimation of Proline and Sugars Contents

The proline concentrations were assayed in ‘Kinnow’ mandarin treated and un-treated leaves at 520 nm [54]. The soluble sugars content was examined by using the method of Gurrieri et al. [55]. A sample of fresh leaves (0.5 g) of treated and untreated ‘Kinnow’ mandarin plants in test tubes containing 10 mL of 80% ethanol were kept in a water bath for 1 h. Next, 0.5 mL of the sample extract was mixed with 1 mL of 18% phenol and kept at room temperature for 1 h. After this 2.5 mL of sulphuric acid was mixed with the mixture and the absorbance was measured at 490 nm. The soluble sugar content was measured with the help of the given formula:

Sugar (μg g^−1^ FW) = Absorbance of plant sample × Dilution factor × K value/Fresh plant tissue weight (7)

#### 2.6.7. Malondialdehyde (MDA) and Hydrogen Peroxide (H_2_O_2_) Content

MDA content was estimated in the leaves of treated and untreated ‘Kinnow’ mandarin plants by using a spectrophotometer at 532 nm [56]. The reaction of H_2_O_2_ with potassium iodate was quantified spectrophotometrically at 390 nm [57] to quantify the H_2_O_2_ content in ‘Kinnow’ mandarin leaves collected from each treatment with the help of the H_2_O_2_ standard curve.

### 2.7. Statistical Analysis

Analysis of Variance (ANOVA) was used for the statistical analysis of the results, and the significance between treatments was evaluated by Duncan’s multiple range test at 0.05% by using statistical software (version 18; Armonk, NY, USA).

## 3. Results

### 3.1. PCR Based Detection of Candidatus Liberibacter spp. in HLB-Infected ‘Kinnow’ Mandarin Plants

**Figure 1:**
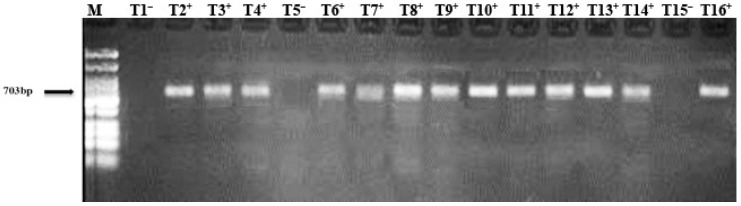
Polymerase chain reaction (PCR) product of 703 bp amplified with forward and reverse primers A2/J5 on 1.5% agarose gel. HLB-infected ‘Kinnow’ mandarin plants (+) and healthy plants (–) and M (100 bp DNA ladder).

The specific set of forward and reverse primers A2/J5 were used for the amplification of the *Liberibacter* ribosomal protein gene and a band of 703 bp was acquired from the HLB-infected ‘Kinnow’ mandarin trees. Figure 1 clearly demonstrates that with the amplified DNA on a 1.5% agarose gel, a band of 703 bp was obtained from HLB-infected ‘Kinnow’ mandarin trees; however, there was no amplification from healthy samples.

### 3.2. Sequencing Analysis

**Figure 2:**
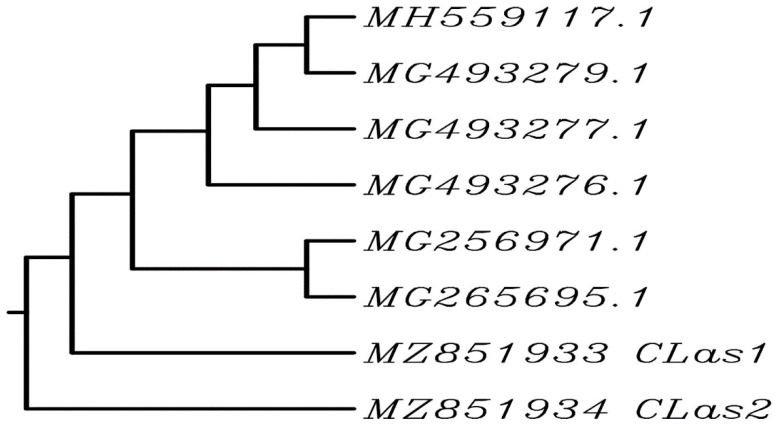
Phylogenetic tree showing the homology of the identified bacterial strain with different nucleotide sequences from the GenBank database (https://www.ncbi.nlm.nih.gov/ (accessed on 13 December 2021)).

BLAST was used to analyze the nucleic acid sequence similarity of local isolates from Pakistan (CLas1 and CLas2 with accession numbers MZ851933 and MZ851934, respectively) and six different sequences from the GenBank database (accession numbers, MH559117, MG256971, MG265695, MG493279, MG49327, and MG493276). The results indicated 100% similarity between MZ851933 (CLas1) and accession number MG265695 (Figure 2).

### 3.3. Morphological and Optical Characterization of Plant Mediated Selenium Nanoparticles (SeNPs)

**Figure 3:**
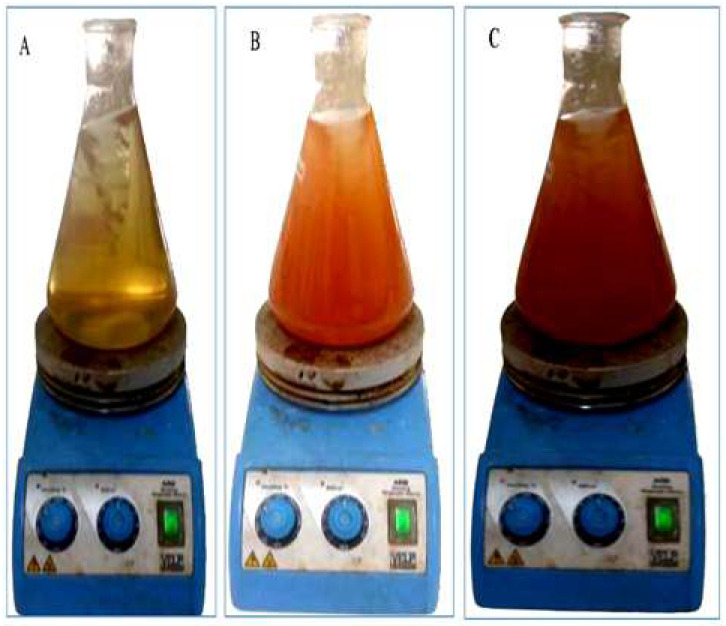
Visual confirmation of garlic clove extract-mediated SeNPs. (**A**) Change in the reaction mixture before the synthesis of nanoparticles. (**B**,**C**) Change in the reaction mixture after the synthesis of SeNPs.

**Figure 4:**
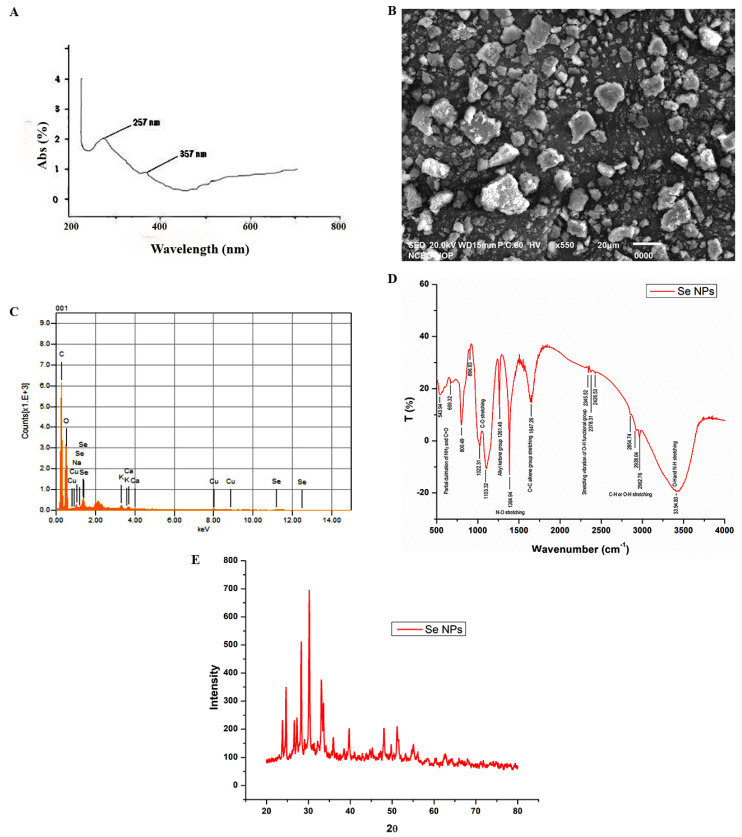
Morphological and optical characterization of biofabricated SeNPs. (**A**) UV-visible spectrum, (**B**) SEM image, (**C**) elemental composition analysis, (**D**) Fourier transform infrared spectroscopy (FTIR), and (**E**) X-ray diffraction analysis (XRD).

Plant extract-mediated green nanomaterial synthesis has advantages over routinely used chemical and physical fabrication methods. In this research, the SeNPs formulation was detected by various characterization techniques. The ongoing research reports the green synthesis of SeNPs by using the garlic clove extract of *Allium sativum* L. as a reducing, capping, and stabilizing agent. The change in color to brick red was the first indication of the fabrication of SeNPs (Figure 3). The SeNPs synthesis was confirmed by using the UV-visible spectrum within the range of 200–800 nm. The results revealed that the characterization peak for the biofabricated SeNPs was in the range of 200 to 400 nm. However, the characterization absorption peak was acquired at 357 nm and another peak at 257 nm, indicating the surface plasmon resonance characteristics of plant-mediated SeNPs (Figure 4A).

The structural quantification of plant-mediated SeNPs was monitored by using scanning electron microscopy. Scanning electron microscopic images of green-synthesized SeNPs exhibited that these particles are spherical, cylindrical, or rectangular in shape (Figure 4B). However, some of these were anisotropic and irregular in shape. Most of the nanostructures were observed at a size of 40–100 nm. The elemental composition of plant-mediated SeNPs was examined through energy-dispersive X-ray analysis. The EDX spectroscopic analysis reveals the confirmation of the purity and existence of SeNPs. The maximum characterization peaks of selenium ranged from 2.5 to 3.5 Kev (Figure 4C). XRD analysis (Figure 4E) and prominent peaks in the pattern were clearly observed. The XRD analysis of the SeNPs revealed the amorphous nature of selenium nanoparticles and very well support the JCPDS card no. 06-0362.

The FTIR analysis is provided in Figure 4D. Our results revealed that absorption peaks of 3200–3400 cm^−1^ can be due to the presence of NH and OH that are involved in the biogenesis of SeNPs. The peak at 2854.74 cm^−1^ may be due to carbon and hydrogen stretching. Additionally, the absorption peak at 1647.26 cm^−1^ can be assigned as C=C alkene group stretching. Furthermore, the absorption peak at 1022.31 cm^−1^ suggests the presence of C-O stretching, which may be attributed to the alcohol group, which denotes that garlic extract interacts with sodium selenite. The absorption peak around 1384.94 cm^−1^ may be attributed to the presence of oxygen and nitrogen groups. Peaks around 500 to 700 cm^−1^ may be attributed to the partial deuteriation of carboxylic or amine groups. Peaks around 2900 cm^−1^ show the presence of aldehyde groups. The presence of various functional groups in garlic clove extract indicates that these are involved in reducing sodium selenite salts in green SeNPs (Figure 4D). Similar results were presented in some other studies indicating the involvement of C=C, O-H, N-H, and C=O groups in the synthesis of SeNPs [38]. In addition, it is also reported in some other studies that N-H, C-H, and C=O functional groups are responsible for the synthesis of SeNPs [38,58].

### 3.4. Differential Impacts of Plant Mediated SeNPs on Physiological Profiling of HLB-Infected ‘Kinnow’ Mandarin Plants

**Figure 5:**
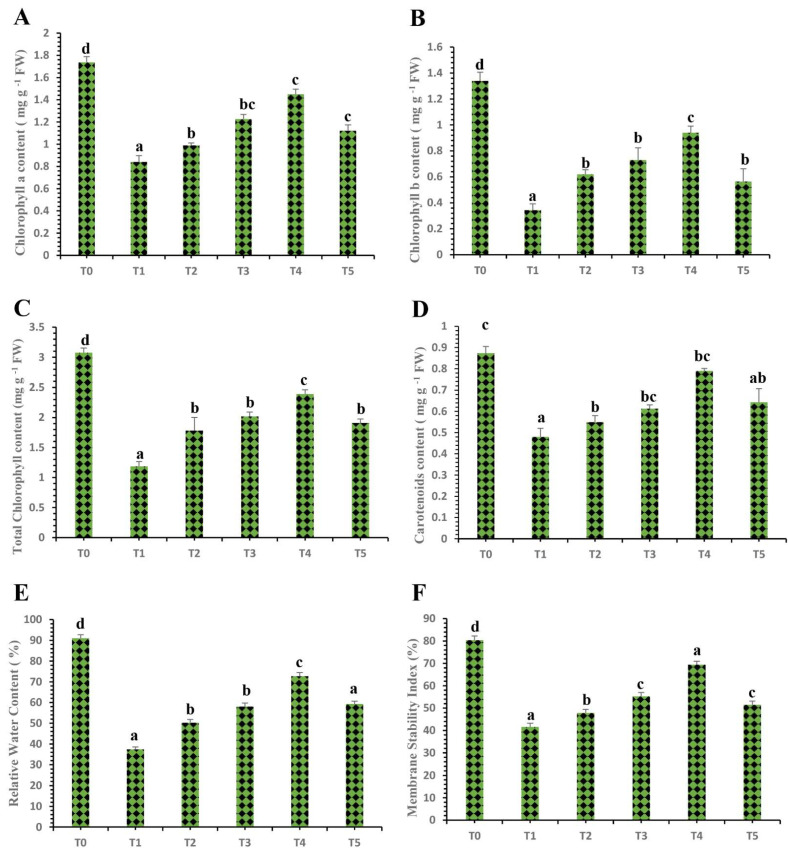
Differential effects of plant-based selenium nanoparticles on (**A**) chlorophyll a, (**B**) chlorophyll b, (**C**) total chlorophyll, (**D**) carotenoid contents, (**E**) relative water content, and (**F**) membrane stability index. Treatments: T0, control healthy ‘Kinnow’ mandarin plants; T1, HLB-infected ‘Kinnow’ mandarin plants; T2, HLB-infected ‘Kinnow’ mandarin plants with SeNPs at 25 mg L^−1^; T3, HLB-infected ‘Kinnow’ mandarin plants with SeNPs at 50 mg L^−1^; T4, HLB-infected ‘Kinnow’ mandarin plants with SeNPs at 75 mg L^−1^; and T5, HLB-infected ‘Kinnow’ mandarin plants with SeNPs at 100 mg L^−1^. Different letters above the column indicate that the results are significantly different (*p* < 0.05).

The physiological parameters of ‘Kinnow’ mandarin plants were investigated to analyze the potential of plant-mediated SeNPs against HLB-infected ‘Kinnow’ mandarin plants. The current results showed that HLB disease has decreased the chl a, b total chl, and carotenoids by 51.44%, 74.62%, 61.56%, and 44.82%, respectively, compared to the control healthy plants (Figure 5). However, the results revealed that the SeNPs have differential impacts on the HLB-infected ‘Kinnow’ mandarin plants. The results showed that SeNPs at 25 mg L^−1^ increased the chl a, chl b, total chl, and carotenoid contents by 18.21%, 12.35%, 33.70%, and 14.58%, respectively, as compared to untreated diseased ‘Kinnow’ mandarin plants (Figure 5). Similarly, SeNPs at 50 mg L^−1^ improved chl a, b, total chl, and carotenoid contents by 45.95%, 53.42%, 39.48%, and 27.08%, respectively, as compared to the untreated HLB-infected ‘Kinnow’ mandarin plants (Figure 5). Surprisingly, SeNPs at 75 mg L^−1^ showed an outstanding improvement in chl a, chl b, total chl, and carotenoid contents by 72.61%, 65.66%, 50.62%, and 64.58%, respectively, as compared to untreated diseased plants (Figure 5). Moreover, the results showed that SeNPs at 100 mg L^−1^ improved the chl a, b, total chl, and carotenoid contents by 33.32%, 14.70%, 42.37%, and 33.33%, respectively, compared to untreated diseased plants (Figure 5).

Similarly, the membrane stability index (MSI) and relative water contents (RWC) are essential factors necessary for the normal physiological functioning of plants. Surprisingly, SeNPs also showed differential impacts on HLB-infected ‘Kinnow’ mandarin plants. HLB disease decreased the MSI and RWC contents by 48.18% and 58.98%, respectively, compared to the control healthy ‘Kinnow’ mandarin plants. However, the obtained results from the current study revealed that SeNPs ameliorated MSI and RWC contents gradually, with SeNPs at 75 mg L^−1^ found to be most effective to enhance the MSI and RWC contents remarkably (Figure 5). The results showed that the SeNPs at 75 mg L^−1^ increased the MSI and RWC contents by 66.81% and 70.53%, respectively, as compared to untreated HLB-infected ‘Kinnow’ mandarin plants. However, the MSI and RWC contents tend to decrease as the concentration of plant-based SeNPs increases to 100 mg L^−1^.

**Figure 6:**
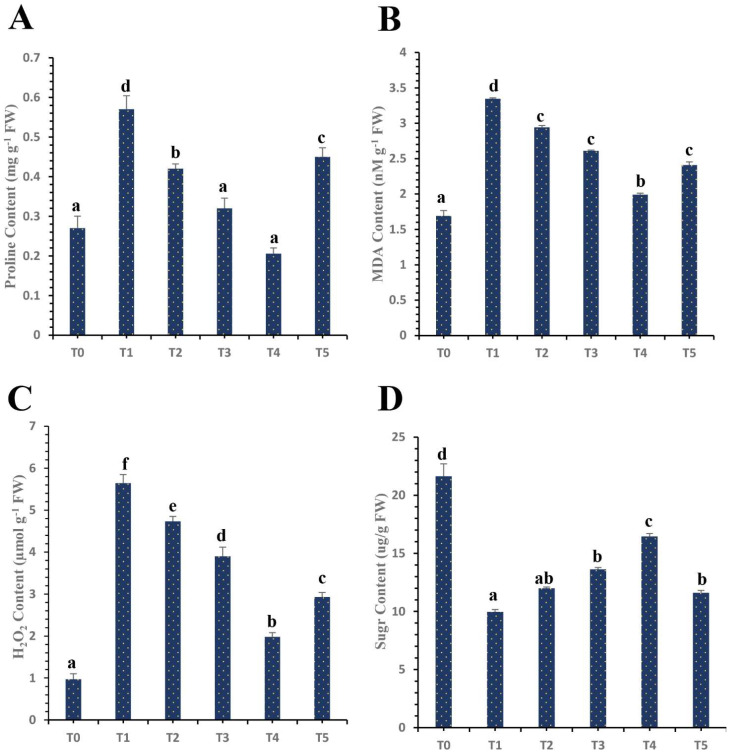
Differential effects of treatments of SeNPs on (**A**) proline content, (**B**) malondialdehyde (MDA) content, (**C**) hydrogen peroxide (H_2_O_2_) content, and (**D**) sugar content. Treatments: T0, control healthy ‘Kinnow’ mandarin plants; T1, HLB-infected ‘Kinnow’ mandarin plants; T2, HLB-infected ‘Kinnow’ mandarin plants with SeNPs at 25 mg L^−1^; T3, HLB-infected ‘Kinnow’ mandarin plants with SeNPs at 50 mg L^−1^; T4, HLB-infected ‘Kinnow’ mandarin plants with SeNPs at 75 mg L^−1^; and T5, HLB-infected ‘Kinnow’ mandarin plants with SeNPs at 100 mg L^−1^. Different letters above the column indicate that the results are significantly different (*p* < 0.05).

HLB disease leads to the enhanced production of proline in infected ‘Kinnow’ mandarin plants. Proline content was remarkably elevated under biotic stress by 96.32% as compared to the control healthy ‘Kinnow’ mandarin plants. However, our results showed that plant-mediated SeNPs have differential impacts on the production of proline content. The results revealed that proline content was decreased by 26.19% when SeNPs at 25 mg L^−1^ were exogenously applied on HLB-infected ‘Kinnow’ mandarin plants. However, the foliar spray of SeNPs at 50 mg L^−1^ decreased the proline content by 39.11% as compared to untreated HLB-diseased ‘Kinnow’ mandarin plants. Surprisingly, our results showed that SeNPs at 75 mg L^−1^ decreased the proline content by 70.96%. However, it was observed that the proline increased when the concentration of SeNPs increased to 100 mg L^−1^ (Figure 6).

Moreover, the soluble sugars content was analyzed in treated and untreated ‘Kinnow’ mandarin plants. Sugars are very vital osmolytes that help in the defensive mechanism of plants against biotic stress. According to our experimental research results, HLB disease drastically affects the soluble sugar content in plants. The obtained results showed that the soluble sugar content decreased by 54.01% in diseased plants as compared to healthy plants. However, according to the obtained results, the application of SeNPs increased the total sugar content significantly in diseased plants as compared to untreated ‘Kinnow’ mandarin plants. The best results were obtained with the application of SeNPs at 75 mg L^−1^, which increased the sugar content by 65.16% as compared to untreated infected plants. However, the sugar content declined as the SeNPs concentration was increased to 100 mg L^−1^ (Figure 6).

### 3.5. Differential Effects of Plant Mediated SeNPs on H_2_O_2_ Content and MDA Content of HLB-Diseased ‘Kinnow’ Mandarin Plants

H_2_O_2_ and MDA concentrations were significantly increased by 82.80% and 97.63%, respectively, in HLB-diseased ‘Kinnow’ mandarin plants as compared to the healthy control plants. However, our results revealed that SeNPs noticeably decreased the H_2_O_2_ and MDA in infected plants. According to the current results, SeNPs at 25 mg L^−1^ reduced the H_2_O_2_ and MDA levels by 16.13% and 11.97%, respectively, in HLB-diseased ‘Kinnow’ mandarin plants (Figure 6). Moreover, SeNPs at 50 mg L^−1^ reduced these levels by 30.85% and 21.85%, respectively, in HLB-diseased ‘Kinnow’ mandarin plants. Interestingly, remarkable results were obtained with the application of SeNPs at 75 mg L^−1^, which decreased H_2_O_2_ and MDA by 64.89% and 40.41%, respectively, compared to untreated HLB-diseased ‘Kinnow’ mandarin plants (Figure 6). The current results showed that the level of H_2_O_2_ and MDA starts to increase as the concentration of SeNPs increases to 100 mg L^−1^ (Figure 6).

### 3.6. Differential Impacts of Plant Mediated Selenium Nanoparticles on Antioxidant Enzymatic and Non-Enzymatic Activity

**Figure 7:**
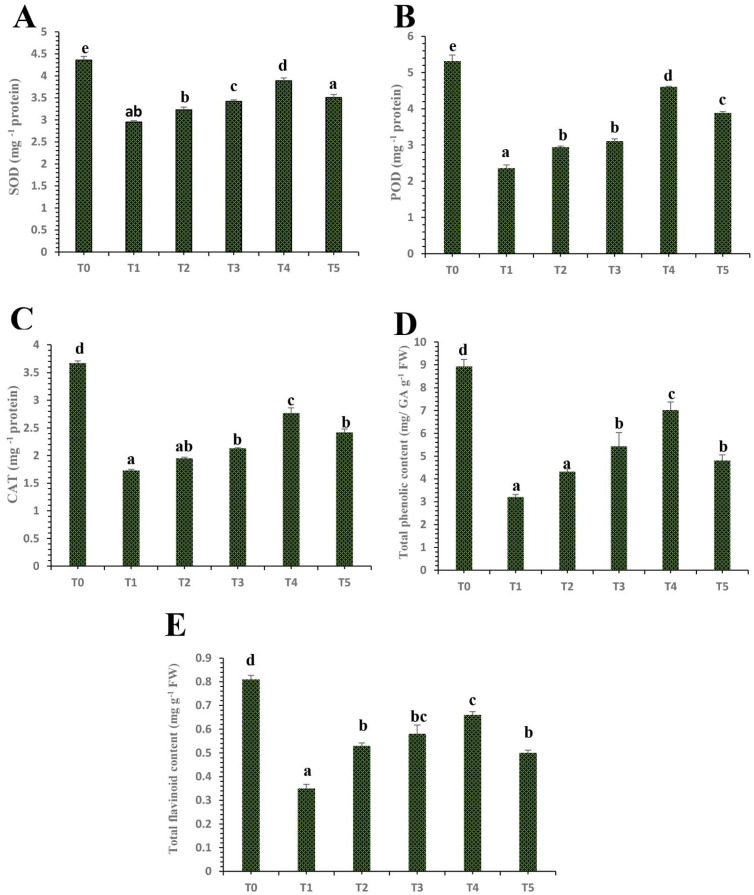
Differential effects of treatments of SeNPs on (**A**) SOD content, (**B**) POD content, (**C**) CAT content, (**D**) total phenolic content, and (**E**) total flavonoid content. Treatments: T0, control healthy ‘Kinnow’ mandarin plants; T1, HLB-infected ‘Kinnow’ mandarin plants; T2, HLB-infected ‘Kinnow’ mandarin plants with SeNPs at 25 mg L^−1^; T3, HLB-infected ‘Kinnow’ mandarin plants with SeNPs at 50 mg L^−1^; T4, HLB-infected ‘Kinnow’ mandarin plants with SeNPs at 75 mg L^−1^; and T5, HLB-infected ‘Kinnow’ mandarin plants with SeNPs at 100 mg L^−1^. Different letters above the column indicate that the results are significantly different (*p* < 0.05).

The activities of enzymatic and non-enzymatic antioxidants such as superoxide dismutase (SOD), POD, catalase (CAT), total phenolic content (TPC), and total flavonoids content (TFC) were significantly increased by the various concentrations of plant-mediated SeNPs (Figure 6). However, the results showed that HLB disease devastated the abovementioned enzymatic and non-enzymatic activities (SOD, POD, CAT, TPC, and TFC) by 32.33%, 55.66%, 52.62%, 64.02%, and 56.79%, respectively, as compared to the control healthy ‘Kinnow’ mandarin plants (Figure 7). The results showed that plant-mediated SeNPs showed differential impacts on HLB-infected ‘Kinnow’ mandarin plants to mitigate the adverse effects by modifying the antioxidant, enzymatic, and non-enzymatic activities. The results showed that the application of SeNPs at 25 mg L^−1^ increased the level of SOD, POD, CAT, TPC, and TFC contents by 8.48%, 19.72%, 11.13%, 25.72%, and 33.77%, respectively, as compared to untreated diseased ‘Kinnow’ mandarin plants (Figure 7). However, the results demonstrated that SeNPs at 50 mg L^−1^ enhanced the level of enzymatic and non-enzymatic antioxidant potential by 13.74%, 24.11%, 18.49%, 40.86%, and 39.45%, respectively as compared to untreated infected ‘Kinnow’ mandarin plants (Figure 7). The obtained results showed that SeNPs at 75 mg L^−1^ showed remarkable results and was found to be the best concentration of SeNPs for enhancing the antioxidant defense system of HLB-infected ‘Kinnow’ mandarin plants by upregulating enzymatic and non-enzymatic activities. The results showed that the enzymatic and non-enzymatic activities were enhanced by 24.01%, 48.80%, 37.28%, 54.23%, and 46.99%, respectively, when infected plants were treated with SeNPs at 75 mg L^−1^. However, our results demonstrated that the level of enzymatic and non-enzymatic activities decreased as the concentration of SeNPs increased to 100 mg L^−1^ (Figure 7).

## 4. Discussion

### 4.1. Identification of HLB Disease in ‘Kinnow’ Mandarin

The detection of HLB disease in ‘Kinnow’ mandarin plants is important to prevent this disease’s widespread outbreak. We identified the HLB-diseased ‘Kinnow’ mandarin plants in the Sargodha district by using the conventional PCR method (Figure 1). The PCR assay is a reliable method for the detection of fastidious bacteria in their host. However, the PCR results follow the findings of Hussain et al. [10] and Ruangwong and Akarapisan [59]. Additionally, the partial sequence of the ribosomal protein gene obtained after the amplification with forward and reverse primers A2/J5 were similar to the existent sequencing in NCBI, and when these sequences were compared with already-known Candidatus Liberibacter species in the gene bank, the similarity percentage was 100% with *Candidatus* Liberibacter asiaticus bacteria (Genbank accession numbers MH559117.1, MG256971.1, MG265695.1, and MG493279.1) (Figure 2). This study confirms the presence of the HLB disease in ‘Kinnow’ mandarin plants selected for the experiment.

### 4.2. Differential Effects of Plant Mediated SeNPs on Physiological Profiling of HLB-Infected ‘Kinnow’ Mandarin Plants

After confirming HLB-infected ‘Kinnow’ mandarin plants, various concentrations of plant extract-mediated SeNPs were used to check physiological and biochemical modifications in HLB-positive ‘Kinnow’ mandarin plants. To obtain deep insights on the impact of plant extract-mediated SeNPs on HLB-affected plants, a series of physiological analyses were performed on chlorophyll contents, carotenoid contents, membrane stability index (MSI), relative water contents (RWC), and proline (PRO) contents. The photosynthesis rate in plants is determined by the chlorophyll and carotenoid contents. Moreover, chlorophyll and carotenoid contents are essential for plants’ photosynthesis and are contained in photosystems I and II. Phytopathogenic bacteria can negatively affect the photosynthetic potential of plants, causing necrosis and resulting in a reduction in chlorophyll contents. Biotic stress leads to severe oxidative damage and ROS production in plants leads to the destruction of the photosynthetic machinery and ultimately to plant death [21,60,61,62]. Therefore, maintaining the chlorophyll and carotenoid contents in plants is essential when they are attacked by various pathogens, as this will allow the plants to continue to perform their photosynthesis phenomenon. The obtained results from the current study showed that HLB disease decreases the chlorophyll a, chlorophyll b, total chlorophyll, and carotenoid contents in citrus plants. The foliar applications of SeNPs significantly ameliorated the chlorophyll and carotenoid contents in HLB-infected plants as compared to untreated diseased ‘Kinnow’ mandarin plants (Figure 5). The highest chlorophyll and carotenoid contents were observed in the HLB-infected plants treated with an application of SeNPs at a concentration of 75 mg L^−1^. However, a decrease in chlorophyll and carotenoid contents was observed when the concentration of SeNPs was increased to 100 mg L^−1^.

There is significantly little data on SeNPs treatments on plants under biotic stresses. The current results pronounced regarding the significant amelioration of chlorophyll and carotenoid contents are in accordance with the findings of Quiterio-Gutiérrez et al. [62] and Zahedi et al. [63], who reported similar results and demonstrated that SeNPs increased the chlorophyll contents of tomato plants under stress by *Alternaria solani*. Furthermore, Dong et al. [64] showed that SeNPs significantly enhanced the chlorophyll and carotenoid contents by 200–400% in *Lycium chinense* L. leaves. Moreover, Ragavan, Ananth, and Rajan [20] reported that SeNPs increased the photosynthetic pigments in cluster beans. An earlier study also suggested that selenium might accelerate chlorophyll biosynthesis by facilitating respiration and electron transport in the respiratory chain [65]. Therefore, an increase in chlorophyll contents in SeNPs-treated HLB-infected plants might contribute to the restoration of photosynthetic machinery and subsequent growth properties. Moreover, selenium nanoparticles protect the structure of chloroplast under severe oxidative damage such as the destruction of stroma lamella and grana lamellae and accelerate the photosynthetic machinery’s biosynthesis by protecting the chloroplast enzymes [66]. The decrease in chlorophyll and carotenoid contents when HLB-infected plants were treated with the application of SeNPs at a concentration of 100 mg L^−1^ might be due to many reasons, such as the generation of ROS, which cause oxidative damage to the plant. It has also been reported that high amounts of selenium interface with amino acids having selenium and sulfur metabolisms and displace sulfur amino acids and proteins synthesized by them, which alter plants’ physiological and biochemical profiling [67].

Biotic stresses significantly decrease plants’ membrane stability and relative water content. Low water availability leads to turgor pressure loss in the plants, checking the cells’ expansion and disturbing plant morphology, physiology, and biochemistry. The generation of ROS destroys the structural integrity of the plasma membrane and causes electrolyte leakage. The concentration of RWC content depends on the uptake of water and the transpiration rate in plants [68]. The results demonstrated that biotic stress significantly decreased the RWC and MSI contents in HLB-diseased ‘Kinnow’ mandarin plants compared to the healthy control plants. However, foliar applications of plant extract-mediated SeNPs increased the amount of RWC and MSI (Figure 5). It is reported that selenium nanoparticles enhance the antioxidant defense system and decrease the level of ROS species in plants, which improves relative water content and increases cell membrane integrity [69]. Additionally, the obtained results from the current study agree with the findings of Zahedi et al. [70], who stated that the selenium and silicon nanostructures increased the amount of RWC and maintained the integrity of the plasma membrane under stress conditions. There is limited research exploring the potential of plant-based selenium nanoparticles to cope with biotic stresses in plants. However, some other studies also showed that plant-mediated titanium dioxide nanoparticles increased the level of relative water content and improved the integrity and stability of wheat plants under biotic stress [68].

### 4.3. Differential Effects of Plant Mediated SeNPs on Antioxidant Defensive Enzymes Activities of HLB-Diseased ‘Kinnow’ Mandarin Plants

The immune system is critical for keeping plants and animals healthy from devastating pathogens. However, immune-related diseases are prevalent in animals, but these are rarely explored for plants. Recently, a published study was reported that HLB disease is an immune-related disease devastating the antioxidant defense system of citrus plants [71]. Similarly, in our research study, the results revealed that HLB disease significantly decreased the level of defensive enzymes such as CAT and POD as compared to the control healthy citrus plants [10]. The obtained results showed that exogenously foliar applications of green-synthesized SeNPs increased the level of the above-mentioned defense-related enzymes in HLB-infected ‘Kinnow’ mandarin plants as compared to untreated HLB-infected ‘Kinnow’ mandarin plants (Figure 7). According to the obtained results, the application of SeNPs at a concentration of 75 mg L^−1^ was perfect for enhancing the level of antioxidant enzymes. The current study results are in-line with the findings of Quiterio-Gutiérrez et al. [62], who reported similar results and demonstrated that selenium and copper nanoparticles increase the level of enzymes in tomato plants suffering from biotic stress. There is limited research regarding the usage of SeNPs against biotic stresses in plants. However, it has been reported that the application of SeNPs increased the level of SOD and CAT enzymes in sorghum plants subjected to stress, which increases stress tolerance [72]. Similarly, many other studies also reported that the application of selenium nanoparticles induced the activities of POD and SOD in strawberry plants subjected to stress [63].

The findings of this study are also supported by the study of Jiang et al. [73] who reported that selenium applications induced the antioxidant defense genes and increased the concentration of CAT and SOD in maize, leading to increased stress tolerance in plants. Furthermore, our results are also in line with previous findings of Khalifa et al. [74], who reported a selenium-mediated antioxidant defense response in plants. Our results also revealed that selenium nanoparticles decreased the concentration of the antioxidant defense-related enzymes mentioned above at 100 mg L^−1^. The toxicological impacts of a high concentration of selenium nanoparticles might be due to the increased level of selenium because it is reported in many published studies that selenium at a high level acts as a pro-oxidant and leads to drastic effects on plants. Additionally, our results are also supported by the study of Tarrahi et al. [75], who reported the toxicological effects of selenium nanoparticles on Lemna minor. The primary toxicological mechanisms of nanoparticles are the release of ROS and oxidative stress generation [22].

### 4.4. Differential Effects of Plant Mediated SeNPs on H_2_O_2_ Content and MDA Content of HLB-Diseased ‘Kinnow’ Mandarin Plants

It is well established that stress conditions cause severe oxidative damage to plants due to the accumulation of elevated levels of ROS, resulting in uncontrolled oxidative cascades that destroy essential cellular components of plants and eventually cause cell death. It has been reported that biotic and abiotic stresses increase MDA and H_2_O_2_ in plants [76,77]. Similarly, the results revealed that the HLB disease increased the level of MDA and H_2_O_2_ contents in the citrus plants as compared to the control healthy ‘Kinnow’ mandarin plants. The foliar applications of plant-mediated SeNPs at 75 mg L^−1^ significantly decreased the level of MDA and H_2_O_2_ contents in HLB-diseased ‘Kinnow’ mandarin plants. The maximum decreased MDA and H_2_O_2_ contents under biotic stress could be attributed to the elevated anti-oxidative enzymes, including POD and SOD [78]. It has been reported in many studies that selenium nanoparticles act as an antioxidant under stress conditions and activate the antioxidant defense mechanism [72]. The increase in antioxidant metabolite production by using SeNPs causes a reduction in the level of MDA and H_2_O_2_ contents in plants under stress conditions. The current results are in line with Zahedi et al. [63], who reported that SeNPs decreased the MDA and H_2_O_2_ contents in plants under a stressful environment. However, the toxicological effects of plant-mediated SeNPs at 100 mg L^−1^ might be due to the fact that selenium at an elevated level causes selenosis and severe oxidative stress [21,79].

### 4.5. Differential Effects of Plant-Mediated SeNPs on Proline and Total Sugars Content of HLB-Infected ‘Kinnow’ Mandarin Plants

A stressful environment significantly effects the production of total sugars and proline contents in plants. Proline production plays a vital role in normalizing stress conditions. Moreover, proline helps the plants provide protection from oxidative damage and maintain an osmotic environment. Proline also helps to protect the proteins from denaturation under adverse conditions [80]. Similarly, soluble sugars play a remarkable role in plants’ defensive mechanisms. Sugars constitute the primary substrate providing structural material and energy for defense responses in plants. Moreover, sugar content also acts as signal molecules interacting with hormonal signaling and regulates the plant immune system [81]. According to the current results for the field experiment, HLB disease increases proline content in the ‘Kinnow’ mandarin plants compared to the control healthy ‘Kinnow’ mandarin plants. This is because plants produce proline under stress conditions to cope with the unfavorable effects of biotic stresses. However, the outcome results from the current experiment showed that plant extract-mediated SeNPs decreased the proline contents in HLB-diseased ‘Kinnow’ mandarin plants. However, it has been reported in other studies that plant-mediated titanium dioxide nanoparticles decreased the level of proline contents to normalize the plants’ functioning under biotic stress [68]. Similarly, the obtained results are also in-line with Iqbal et al. [82], who reported that proline contents increase in plants under stress conditions. However, plant-mediated silver nanoparticle exposure decreased the proline contents, which means the stress was alleviated. The results are also supported by the study of Sultana et al. [83], who reported that biotic stress increased the proline content in the plant; however, the foliar applications of plant-mediated silver nanoparticles reduced the proline content in plants under biotic stress. According to the obtained results, HLB disease reduces the level of total sugars in ‘Kinnow’ mandarin plants; however, the application of SeNPs increased the level of total sugars. The results are in agreement with the findings of Zahedi et al. [63], who stated that 20 mg L^−1^ of selenium nanoparticles enhanced the production of osmolytes in strawberry plants under stress conditions.

### 4.6. Differential Effects of Plant Mediated SeNPs on Non-Enzymatic Antioxidant Activities of HLB-Infected ‘Kinnow’ Mandarin Plants

**Figure 8:**
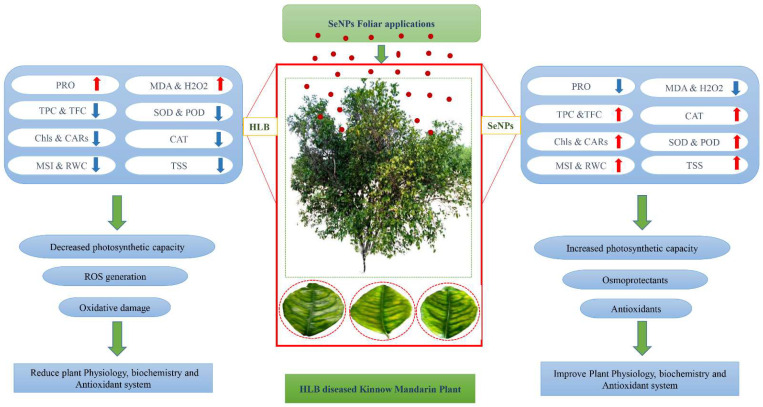
Schematic diagram presented the possible mechanistic action of plant extract mediated selenium nanoparticles (SeNPs) in HLB-infected ‘Kinnow’ mandarin plants. Foliar application exposure of plant-based SeNPs to HLB-diseased Mandarin Kinnow plants could ameliorate the physiological, biochemical, and antioxidant performance by (i) enhancing photosynthetic capacity (ii) activating the antioxidant defensive enzymatic and non-enzymatic activities for maintenance of reactive oxygen species. Relative water content, RWC; total soluble sugars, TSS; chlorophylls, Chls; membrane stability index, MSI; Proline, PRO; malondialdehyde, MDA; hydrogen peroxide, H_2_O_2_; catalases, CAT; superoxide dismutase, SOD; peroxidases, POD; total phenolic content, TPC; total flavonoid content, TFC.

Various biotic and abiotic stresses in plants lead to the disruption of the electron transport chain which is involved in the production of ROS, which are strong oxidizing agents with deleterious effects on plant cells [84]. Plants defend themselves from the drastic effects of ROS through the production of enzymatic and non-enzymatic compounds, which eliminate the ROS and ultimately protect cells from oxidative damage [85]. The production of vital non-enzymatic antioxidants such as flavonoids and phenolic contents is a natural response of plants against stress [86]. It has been reported in many studies that the applications of different nanoparticles can induce the production of antioxidant compounds which can be beneficial to enhance pathogenic resistance in plants [62]. According to our experimental results, HLB disease decreased the level of non-enzymatic compounds such as flavonoids and phenolic compounds in HLB-infected ‘Kinnow’ mandarin plants as compared to control healthy plants (Figure 7). This is because HLB diseases cause the deadliest effects on the antioxidant defense system of plants. However, our results showed that the application of SeNPs enhanced the production of flavonoids and phenolic compounds in HLB-infected plants as compared to untreated HLB-infected ‘Kinnow’ mandarin plants. The current results are in accordance with the findings of Quiterio-Gutiérrez et al. [62], who demonstrated that selenium and copper nanoparticles modify the biochemical profiling by enhancing enzymatic and non-enzymatic antioxidant defense systems in potato plants under biotic stress. There is limited research available to explore the role of selenium nanoparticles in enhancing phenolic and flavonoid contents in plants under biotic stress. However, our results are also supported by the study of Raigond et al. [87], who revealed that zinc nanoparticles increased the contents of phenolic compounds in potato plants. Similarly, The current results also agree with López-Vargas et al. [88], who stated that CuNPs increased the flavonoids content in tomato fruits by 36.14%. The outstanding antioxidant potential of plant-based SeNPs might be due to various phytochemical functional groups that provide capping and stabilizing to nanoparticles and come from primary metabolites [89]. Figure 8 presents the possible mechanistic action of plant extract-mediated selenium nanoparticles (SeNPs) in HLB-infected ‘Kinnow’ mandarin plants.

## 5. Conclusions

In this study, plant-based selenium nanoparticles (SeNPs) have emerged as an excellent biocompatible, eco-friendly approach to overcome the deleterious effects of HLB disease in ‘Kinnow’ mandarin plants. In the current study, we identified the HLB-diseased ‘Kinnow’ mandarin plants and their causative agent *Candidatus* Liberibacter asiaticus by using molecular techniques for the experiment. SeNPs were synthesized using the garlic clove extract of *Allium sativum* L. The optical and morphological techniques confirmed that the synthesized nanoparticles were observed as spherical, cylindrical, or rectangular in shape with a size of 40–100 nm. The obtained results showed that SeNPs at 75 mg L^−1^ were more effective in enhancing the photosynthetic potential, membrane stability index (MSI), relative water content (RWC), and total sugars in HLB-infected ‘Kinnow’ mandarin plants as compared to untreated diseased plants. Moreover, an incredible increase in enzymatic and non-enzymatic antioxidants was also observed in treated plants. Interestingly, a marked decrease in H_2_O_2_, MDA, and proline content was observed in treated diseased plants, reinforcing the importance of plant-mediated SeNPs in alleviating the drastic effects of HLB in citrus. To conclude, the excellent antibacterial potency found for this SeNPs formulation at a 75 mg L^−1^ concentration and short exposure time could represent a real alternative for a still-unsolved problem. Overall, this research experiment’s results contribute to the management and treatment of a novel control approach and present the first biocompatible cure for citrus growers to control HLB more effectively. Finally, it can be concluded that phyto-nanotechnology is an important research area that deserves plant researchers’ attention due to its immense potential applications in agriculture. However, a detailed scientific investigation is required to understand the bridge between selenium and SeNPs. Collaborative efforts from plant biologists, physiologists, pathologists, and nanotechnologists are required to investigate the exact mechanistic action of selenium nanoparticles in in vivo conditions. This will have a significant impact on controlling HLB disease in citrus and help save citrus production worldwide.

## Figures and Tables

**Table 1:** SeNPs treatments plan.

Treatment	Conditions
T0	Control (healthy ‘Kinnow’ mandarin plants)
T1	HLB-infected ‘Kinnow’ mandarin plants (distillted water)
T2	HLB-infected ‘Kinnow’ mandarin plants + 25 mg L^−1^ SeNPs
T3	HLB-infected ‘Kinnow’ mandarin plants + 50 mg L^−1^ SeNPs
T4	HLB-infected ‘Kinnow’ mandarin plants + 75 mg L^−1^ SeNPs
T5	HLB-infected ‘Kinnow’ mandarin plants + 100 mg L^−1^ SeNPs

## Data Availability

All the obtained data are presented in this article.
